# New Frontiers in Open and Minimally Invasive Abdominal Oncologic Surgery

**DOI:** 10.3390/jcm14176168

**Published:** 2025-09-01

**Authors:** Maria Lanzone, Immacolata Iannone, Chiara D’Alterio, Cristina De Padua, Alessandro Coppola

**Affiliations:** Dipartimento di Chirurgia, Sapienza University of Rome, Viale Regina Elena 291, 00161 Rome, Italy; mari.lanzone97@gmail.com (M.L.); immacolata.iannone@uniroma1.it (I.I.); c.depadua@policlinicoumberto1.it (C.D.P.); coppola.chirurgia@gmail.com (A.C.)

In recent decades, the landscape of abdominal oncologic surgery has undergone a transformative shift, largely propelled by remarkable advancements in surgical techniques and integrative treatment modalities [1]. Traditional open surgeries, once the cornerstone of oncologic resection, now share the stage with minimally invasive techniques such as laparoscopic and robotic-assisted procedures. These innovations not only aim to enhance oncologic efficacy but also prioritize patient-centered outcomes such as faster recovery, reduced morbidity, and improved quality of life.

Despite the growing enthusiasm for minimally invasive approaches, open surgery remains indispensable in managing complex oncologic cases. Particularly in tumors requiring multivisceral resections, vascular involvement, or extensive intra-abdominal dissection, the open approach provides surgeons with maximal tactile feedback, exposure, and control. Such scenarios are commonly encountered in advanced gastrointestinal, hepatobiliary, and pancreatic malignancies, where complete tumor clearance is paramount for improving survival outcomes [2,3]. While technological advances continue to redefine boundaries, open surgery retains its status as the gold standard for complex abdominal oncologic procedures.

Laparoscopic surgery has revolutionized abdominal oncology by offering patients reduced postoperative pain, shorter hospital stays, and quicker return to daily activities. In colorectal cancer surgery, for instance, multiple randomized controlled trials and meta-analyses have demonstrated that laparoscopic resection achieves comparable oncologic outcomes to open surgery, with added benefits in terms of postoperative recovery [4]. Similarly, for early-stage gastric cancer, laparoscopic gastrectomy is now widely accepted as an effective alternative to open procedures [5]. The continued evolution of laparoscopic tools, including articulating instruments and high-definition imaging, is pushing the envelope further, allowing for more complex resections with oncologic and overall safety.

Robotic-assisted surgery has emerged as a game changer in abdominal oncology. By enhancing dexterity, visualization, and surgeon ergonomics, the robotic platform addresses several limitations of conventional laparoscopy. Its utility is particularly evident in confined anatomical spaces, such as the pelvis or retroperitoneum, and in complex procedures like pancreaticoduodenectomy or technically demanding hepatic resections [6,7].

The three-dimensional magnified view and articulating instruments of robotic systems facilitate meticulous dissection and reconstruction, which is crucial in oncologic surgery to ensure negative margins and adequate lymphadenectomy. Early studies suggest that robotic approaches may be associated with lower conversion rates, reduced blood loss, and shorter learning curves for certain procedures, although cost-effectiveness remains a topic of ongoing debate [8].

Beyond mechanical innovations, the integration of novel therapeutic strategies such as Hyperthermic Intraperitoneal Chemotherapy (HIPEC) and Pressurized Intraperitoneal Aerosol Chemotherapy (PIPAC) has significantly expanded the armamentarium against peritoneal surface malignancies. HIPEC, which involves the intraoperative circulation of heated chemotherapeutic agents within the peritoneal cavity, has shown promise in selected patients with peritoneal carcinomatosis from colorectal, gastric, or ovarian origins [9,10]. It offers the advantage of delivering high local drug concentrations while minimizing systemic toxicity.

PIPAC represents a newer, minimally invasive technique in which chemotherapeutic agents are aerosolized and applied under pressure within the peritoneal cavity during laparoscopy. This approach enhances drug distribution and penetration into tumor nodules while maintaining low systemic absorption. PIPAC has been shown to improve symptom control and disease stabilization in patients with refractory peritoneal metastases, making it a valuable palliative option and, in some cases, a bridge to cytoreductive surgery with HIPEC [11].

This multidisciplinary approach also underscores the importance of collaboration between surgical oncologists, medical oncologists, anesthesiologists, and radiologists. Precision imaging, enhanced recovery after surgery (ERAS) protocols, and personalized treatment planning are becoming standard components of care, reinforcing the need for a holistic view of the patient journey.

As we move toward an era of precision medicine, the role of artificial intelligence (AI), machine learning, and augmented reality in abdominal oncologic surgery cannot be overstated. From preoperative planning and intraoperative navigation to outcome prediction and postoperative monitoring, digital tools are set to play an increasingly integral role in surgical decision-making [12].

The future of abdominal oncologic surgery lies at the intersection of technical innovation, personalized treatment, and multidisciplinary collaboration. By embracing minimally invasive techniques, enhancing surgical precision through robotics, and integrating effective adjunct therapies like HIPEC and PIPAC, we can offer patients not only improved survival but also a better quality of life.
